# Dual Phosphorylation of Thr175 and Ser176 Is Essential for SnRK1α1 Activation

**DOI:** 10.1111/ppl.70726

**Published:** 2025-12-29

**Authors:** Alejandra Ávila, Aitana López, Jacquelynne Cervantes, Rogelio Rodríguez‐Sotres, Eleazar Martínez‐Barajas, Patricia Coello

**Affiliations:** ^1^ Departamento de Bioquímica, Facultad de Química UNAM Mexico City Mexico; ^2^ Depto. Microbiología e Inmunología, FMVZ UNAM Mexico City Mexico

**Keywords:** phosphorylation, SnAKs, SnRK1 activation, stress

## Abstract

SnRK1 protein kinases play a pivotal role in regulating plant development, growth signaling, and stress responses by managing cellular responses to energy fluctuations. SnRK1 activation was thought to depend mainly on the phosphorylation of threonine at position 175 (Thr175) within the activation loop. However, recent phosphoproteomic studies have identified additional phosphorylation sites. We explored the functional significance of these modifications, focusing on serine at position 176 (Ser176), adjacent to Thr175 in SnRK1α1. Our results reveal that dual phosphorylation of Ser176 and Thr175 is vital for optimal SnRK1 activity. Structural modeling and thermodynamic analyses highlight the critical role of these modifications in optimising substrate positioning and enzymatic efficiency. Furthermore, only the wild‐type SnRK1α1, which can be phosphorylated at both sites, retains full functionality in in vivo experiments with yeast and Arabidopsis. Interestingly, pSer176 exhibits greater stability than pThr175 at various times throughout the day. Mutant proteins with substitutions at these sites (T175A/S176A mutants) accumulate in cytoplasmic aggregates after heat shock, suggesting a strong link between phosphorylation status, protein stability, and SnRK1 degradation pathways.

## 1 Introduction

SnRK1 (SNF1‐Related Protein Kinase 1) plays a pivotal role in the regulation of various aspects of plant growth and development (Morales‐Herrera et al. 2024; Zheng et al. 2024; Ma et al. 2025). Extensive research has highlighted its critical function in plant signaling pathways, particularly in response to biotic and abiotic stresses (Fichtner and Lunn 2021; Zhang et al. 2024).

Structurally, SnRK1 is a highly conserved heterotrimeric complex consisting of three distinct subunits: one catalytic α subunit and two regulatory subunits, designated as β and βγ. These subunits exhibit isoform diversity, especially within the catalytic component, which exists in two forms: SnRK1α1 and SnRK1α2. Notably, SnRK1α1 accounts for most of the enzymatic activity (Zhang et al. 2009; Broeckx et al. 2016).

The activation of SnRK1 is under the control of two complementary mechanisms. The first involves the assembly of the heterotrimeric complex, which enhances SnRK1 activity compared with the catalytic subunit alone (Maya‐Bernal et al. 2017). The second mechanism involves phosphorylation at the activation T‐loop, a process facilitated by upstream kinases SnAK1 and SnAK2, leading to enhanced kinase activity (Shen et al. 2009; Crozet et al. 2010). Recent phosphoproteomic studies and analyses of SnRK1's interaction networks have confirmed the formation of this complex within plant cells (Leene et al. 2022). However, the specific functions of different SnRK1 complexes remain to be fully elucidated. Interestingly, Ramon et al. (2019) successfully complemented a yeast triple mutant by expression of the catalytic subunit alone. According to this observation, the full heterotrimeric complex may not be strictly necessary for certain SnRK1 functions.

Although SnRK1 may operate as an individual catalytic subunit or as part of a complex, its complete activation is dependent on its phosphorylation at specific threonine residues—Thr175 in SnRK1α1 and Thr176 in SnRK1α2. However, this phosphorylation does not always directly correlate with elevated kinase activity, suggesting the existence of additional regulatory mechanisms of SnRK1's functionality (Fragoso et al. 2009; Rodrigues et al. 2013).

Protein regulation through multiple phosphorylation is a common mechanism for plant protein kinases, and SnRK1 may not be an exception. In fact, protein kinases that rely solely on the phosphorylation of a single residue are uncommon, and the involvement of multiple phosphorylated residues is frequent. Research has shown that AMPK and SNF1 are phosphorylated at various residues, such as Serine 173 in AMPK and Serine 211 in SNF1, which have also been identified as phosphorylation sites, although their modification does not always influence the catalytic activity (Martínez‐Barajas and Coello 2019; Aslam and Ladilov 2022). Furthermore, phosphoproteomic analyses in response to auxin, ABA, and circadian signals have identified phosphorylation at both Thr175 and Ser176 in SnRK1α1 within living cells of Arabidopsis (Umezawa et al. 2013; Zhang et al. 2013; Choudhary et al. 2015).

In this study, Thr175 and Ser176 within the recombinant catalytic domain (CD) of SnRK1α1 were individually substituted with alanine (Ala). Although these substitutions did not inhibit the phosphorylation of the adjacent residue, the enzyme's activity remained low in both instances.

Functional assays, including yeast complementation and transient expression in protoplasts using the full‐length kinase, revealed that mutations at either site fail to restore normal function. In line with these observations, structural models of a predicted SnRK1α1 complex with its substrate suggest a phosphorylation requirement of both Thr175 and Ser176 for an effective position of the AMARA peptide within the enzyme's active site, pointing to stabilizing salt bridge interactions potentially relevant to enzyme catalysis.

Measurements of the levels of phosphorylated Thr175 (pThr175) and Ser176 (pSer176) at the start and end of the day and during extended darkness indicated a strong link between these phosphorylation levels and enzyme activity, highlighting the importance of both sites being phosphorylated. While impairment of phosphorylation at either site did not alter the cytoplasmic and nuclear localization of SnRK1α1 under normal conditions, the non‐phosphorylated mutant protein accumulated in larger cytoplasmic granules during heat stress, suggesting a potential defect in protein recycling mechanisms.

## 2 Material and Methods

### 2.1 Plant Materials and Growth Conditions

The *snrk1α1* and *snrk1α2* *Arabidopsis thaliana* mutants were generated by CRISPR‐Cas9 technology according to the protocol established by Xing et al. (2014). In brief, guide RNA (gRNA) sequences specifically targeting exon 2 of *SnRK1α1* and *SnRK1α2* were identified using an online tool (https://chopchop.cbu.uib.no) (Labun et al. 2019; see Table S1 for *gRNA* sequences). Complementary DNA fragments with 4‐nucleotide 5′ overhangs were cloned into the pCambia‐based vector pHSE401 (Addgene # 62201) via Golden Gate cloning. The recombinant vectors were initially transformed into *E. coli* for sequence validation. Verified constructs were subsequently introduced into *Agrobacterium tumefaciens* strain GV3101, which was used to transform *Arabidopsis thaliana* by the floral dip method (Clough and Bent 1998). T1 transformants were selected based on hygromycin resistance, and successive generations were screened using genomic PCR and sequencing around the target site. Homozygous edited plants were confirmed by identifying a single peak in the chromatogram corresponding to the edited DNA sequence (Figure S1).

Wild‐type *Arabidopsis thaliana* ecotype Columbia (Col‐0), *snrk1α1* and *snrk1α2* mutants were grown in soil (sunshine 3, vermiculite, and agrolite in a proportion of 3:1:1) in a growth chamber at 22°C under normal light conditions (12 h light/12 h dark) at a light intensity of 120 μmol photons m^−2^ s^−1^. For specific experimental purposes, plant samples were harvested at various time points: 8 AM and 6 PM, as well as after 24 h of darkness. These samples were immediately frozen in liquid nitrogen and stored at −70°C until further analysis.

### 2.2 Plasmid Construction

The catalytic domains (CDs) of *SnRK1α1* (At3G01090, bp 1–1023) and *SnRK1α2* (At3G29160, bp 1–1026) were cloned into the pGEX4T‐2 vector. Additionally, the full coding sequences (CDS) of SnRK1‐activated kinases *SnAK2* (AT3G45240) and *SnAK1* (AT5G60550) were also cloned into the pGEX4T‐2 vector, following the methodology described by Maya‐Bernal et al. (2017). For site‐directed mutagenesis, pairs of primers were designed to contain the desired mutations, flanked by 10–15 bases of correct sequence on both sides. Coding regions were amplified by PCR using Phusion High‐Fidelity DNA polymerase (Thermo Scientific) from plasmids containing *SnRK1α1* and *SnRK1α2* cDNA. All mutants were cloned into the pGEX4T‐2 vector (Amersham) using *EcoRI/BamHI* restriction sites.

### 2.3 Recombinant Protein Production and Purification

Recombinant proteins were expressed in the *E. coli* strain BL21 (DE3). Cultures were grown in 50 mL of LB broth (Sigma) at 37°C until reaching an OD600 of 0.5–0.8. Protein expression was induced by 0.3 mM IPTG (isopropyl‐D‐thiogalactopyranoside) (VWR amresco) at 25°C for 4 h. Cells were harvested by centrifugation at 6000 ×*g* for 10 min and stored at −80°C until further use. For protein extraction, bacterial pellets were resuspended in 5 mL of wash buffer (PBS 1X, 1% Triton X‐100, 1 mM EDTA, 1 mM DTT, 1 mM PMSF, 1 mM benzamidine) containing 1 mg/mL lysozyme and then incubated at 4°C for 30 min. Cell disruption was achieved through ten 10‐s sonication pulses. Lysates were centrifuged at 10,000 ×*g* for 30 min to obtain soluble protein fractions. The clarified protein extract was loaded onto a 1 mL bed volume of glutathione agarose (Sigma G4510) following the manufacturer's instructions. Bound proteins were eluted using a buffer containing 10 mM reduced glutathione, 1 mM PMSF, and 1 mM benzamidine in Tris buffer at pH 9.5. Finally, purified proteins were dialyzed against a buffer containing 50 mM Tris (pH 8), 50 mM NaCl, 25% v/v glycerol, 1 mM PMSF, and 1 mM benzamidine.

### 2.4 Kinase Assay

To evaluate SnRK1 activity, 2 μg of the kinase domain were activated by incubating them with 0.2 μg of the SnAK2 kinase. The reaction was carried out in a buffer containing 40 mM HEPES (pH 7.5), 5 mM MgCl_2_, 200 μM ATP, 4 mM DTT, 1× phosphatase inhibitors (Sigma) and 1× protease inhibitor cocktail (Sigma) for 1 h at 30°C. The activity assay was then conducted for 6 min at 30°C. In this assay, 5 μL of the pre‐activated catalytic domain was added to a reaction mixture containing 40 mM HEPES (pH 7.5), 5 mM MgCl_2_, 200 μM ATP with 12.5 kBq (γ‐32P) ATP (Perkin Elmer), 200 μM AMARA peptide (AMARAASAAALARRR), 4 mM DTT, 1× phosphatase inhibitors, and 1× protease inhibitor cocktail (Sigma). To measure the incorporation of (γ‐^32^P) ATP, 15 μL of the reaction mixture was spotted onto phosphocellulose paper (Whatman P81, GE Healthcare), washed with 1% phosphoric acid followed by acetone, and quantified via liquid scintillation counting using Ultima GOLD XR (Perkin Elmer).

In addition to using the radioactive protocol for recombinant proteins, the ADP‐Glo Kinase assay kit (Promega) was also employed. In this method, 1–2 μg of total protein was incubated for 60 min at 23°C in 5 μL of kinase buffer containing 40 mM HEPES (pH 7.5), 20 mM MgCl_2_, 200 μM ATP, 4 mM DTT, and 400 μM AMARA peptide. Negative control reactions were conducted simultaneously, excluding the AMARA peptide.

To stop the reaction, 5 μL of ADP‐Glo reagent was added, followed by a 40‐min incubation at 23°C. Subsequently, 10 μL of kinase detection reagent was added, and the mixture was incubated for an additional 60 min at 23°C. Finally, luciferase activity was measured using a microplate luminometer (BMG Labtech).

Kinase activity was expressed as nmol of ATP consumed per minute per mg of protein.

### 2.5 Phosphorylation Assays

SnRK1α1 and SnRK1α2 catalytic domains were phosphorylated by SnAK1 and SnAK2 in a final reaction volume of 25 μL. The assay was conducted in kinase buffer containing 40 mM HEPES‐KOH (pH 7.5), 5 mM MgCl₂, 200 μM ATP, 4 mM DTT, 1× phosphatase inhibitor cocktail (Sigma), 1× protease inhibitor cocktail (Sigma), and 2 μCi of (γ‐^32^P) ATP (Perkin Elmer). The phosphorylation reaction was carried out at 30°C for 1 h, using 2 μg of the catalytic domain of each SnRK1α (1 and 2) and 0.2 μg of SnAK1/2. Following the reaction, phosphorylated proteins were separated via SDS‐PAGE and transferred onto PVDF membranes. The membranes were then exposed to an Imaging Screen HD (Bio‐Rad), and the images were visualized using a Molecular Imager FX imaging system (Bio‐Rad).

### 2.6 Antibody Detection by ELISA

The titer of specific antibodies was determined by an indirect ELISA assay with commercially synthetized phosphorylated serine (pSer176) or phosphorylated threonine (pThr175) peptides as antigens. Initially, 50 μL of each peptide solution (1 μg/mL) prepared in carbonate buffer was added to the wells of an ELISA plate and incubated overnight at 4°C to facilitate antigen binding. Following incubation, the plates were thoroughly washed to remove unbound peptides and blocked with 150 μL of 1% bovine serum albumin (BSA) in phosphate‐buffered saline (PBS) to prevent non‐specific binding. After blocking, 50 μL samples of 2‐fold serial dilutions of antibody serum in PBS with 1% BSA were added to the wells and incubated for 1 h at 37°C to allow antibody–antigen binding. After washing, with an excess of 1% BSA in PBS, bound antibodies were detected using 50 μL of horseradish peroxidase (HRP)‐conjugated goat anti‐rabbit IgG (H + L) secondary antibody (Abcam), diluted at 1:10,000 in PBS. The enzymatic reaction was initiated by adding the substrate TMB (3,3′,5,5′‐Tetramethylbenzidine) and hydrogen peroxide to render a color change upon conversion by HRP. Color development was stopped by adding 30 μL of 0.1 M hydrochloric acid (HCl) per well. Finally, the optical density (OD) was measured at 450 nm using a microplate spectrophotometer reader (Sinergy, Biotek), providing quantitative data on specific antibody titer.

### 2.7 SDS–PAGE and Western Blotting

Soluble proteins were subjected to electrophoresis after mixing with 2× Laemmli buffer. After electrophoresis, the proteins were transferred to a polyvinylidene fluoride (PVDF) membrane, and immunodetected by incubating overnight with the primary rabbit antibodies at 4°C. Antibodies against pThr175, pSer176, or the catalytic domain (CD) were used as a primary antibody. Horseradish peroxidase (HRP)‐conjugated secondary antibodies (Abcam Ab99697) and chemiluminescent detection reagent were used for signal development (BioRad Cat. 1,705,062).

### 2.8 Structural Prediction of SnRK1α1

Structural models of the catalytic domain (residues 1 to 352) of SnRK1α1 were generated using AlphaFold 3 (Abramson et al. 2024), in complex with its reaction products, MgADP and AMARA phosphorylated at residue Ser 7 (AMARA^pSer7^). The SnRK1α1 sequence was analyzed in various phosphorylation states: unmodified, phosphorylated at Thr175 (SnRK1α1‐pThr175), phosphorylated at Ser176 (SnRK1α1‐pSer176), or phosphorylated at both residues (SnRK1α1‐pThr175‐pSer176). The predictive models showed high accuracy, with ptm values exceeding 0.92 for SnRK1α1 and around 0.6 for the AMARA^pSer7^ peptide, ATP, and Mg^2+^. Geometry consistency was ensured using the semi‐empirical Quantum Mechanics (sQM) PM7 method with localized molecular orbitals (LMO) and implicit solvent (COSMO), as implemented in MOPAC 2016 (Řezáč and Stewart 2023). The AlphaFold raw geometry served as a reference to maintain alignment with the initial model. To infer the geometry of the enzyme‐substrate complex, the phosphate group was transferred from AMARA^pSer7^ to ADP to form ATP, followed by geometry optimization of the entire complex. All atoms were treated as free during these optimization procedures. The peptide binding energy was determined by calculating the difference in the heat of formation (∆Hof) between the whole complex and the separate, rigid‐body species. Visualization and geometry analyses were conducted using UCSF Chimera (Pettersen et al. 2004).

### 2.9 Protoplast Transfection

Arabidopsis protoplasts from *snrk1α1* plant were isolated following the protocol of Wu et al. (2009), with modifications as detailed in Ruiz‐Gayosso et al. (2022). The LUC reporter driven by the *DIN6/ASPARAGINE SYNTHASE 1* promoter was previously described by Baena‐González et al. (2007). To construct the *SnRK1α1* effector vector, the pBRN168 plasmid (Addgene #79668) was used as outlined in Trejo‐Fregoso et al. (2022), replacing the *DsRed* fragment with the *SnRK1α1* WT or cDNAs containing mutations to produce T175A, S176A, or combine T175A/S176A protein variants. The *35S::GFP* construct functioned as the transfection control, with protoplasts transfecting only with the *35S::GFP* construct and the LUC reporter serving as negative controls. Luciferase assays were performed following the protocol by Yoo et al. (2007), with luciferase and GFP signals detected using a CLARIOstar microplate reader (BMG Labtech).

For localization studies, the coding sequence of *SnRK1α1*, along with variants containing T175A, S176A, or combined T175A/S176A mutations, was cloned under the *35S* promoter and fused in‐frame with the GFP coding sequence within the pBRN168 plasmid. Protoplast transfection followed the methodology described by Ruiz‐Gayosso et al. (2022). Post‐transfection, protoplasts were incubated for 1 h at either 25°C or 37°C, and GFP fluorescence was captured using a Leica TCS‐SP8 confocal microscope.

### 2.10 Yeast Transformation

In this study, the *Saccharomyces cerevisiae* deletion mutant used is genetically identical to the BY4742 strain (MATα his3∆1 leu2∆0 lys2∆0 ura3∆0), kindly provided by Dr. Roberto Coria from the Instituto de Fisiología Celular, UNAM. Both *SnRK1α1* wild‐type (WT) sequence and various mutated constructs were cloned into the pDB20 plasmid, which contains the strong, constitutive *ALCOHOL DEHYDROGENASE 1* (*ADH1*) promoter.

Yeast transformation was conducted following the standardized LiAc/SS carrier DNA/PEG protocol, as described by Gietz and Schiestl (2007). The transformed strains were cultivated to the exponential phase in minimal synthetic defined medium lacking uracil (SD‐ura) supplemented with 2% (w/v) glucose at 30°C. Drop assays were then performed on SD‐ura plates containing either 2% (w/v) glucose (as a control) or 2% (v/v) sucrose. Transformants were spotted at an OD600 of 1.0 (10^0^) and four 10× serial dilutions were prepared (10^−1^ to 10^−4^). Yeast growth was monitored after 3 days of incubation at 30°C. The assay was independently replicated twice, producing consistent results.

### 2.11 Statistical Analysis

The experimental data were analyzed utilizing GraphPad Prism 9 software and are presented as mean ± SD. Group comparisons were conducted through one‐way ANOVA, with a *p*‐value of 0.05 indicating statistical significance.

## 3 Results

### 3.1 Phosphorylation and Activation of SnRK1

SnAK1 (GRIK2) and SnAK2 (GRIK1) are critical kinases responsible for the phosphorylation and activation of SnRK1α1 and SnRK1α2 in Arabidopsis (Shen et al. 2009; Crozet et al. 2010; Sun et al. 2024). Research suggests that activation can be triggered via phosphorylation, either by the catalytic domain (CD) or by the full‐length kinase (Shen et al. 2009; Crozet et al. 2010; Maya‐Bernal et al. 2017; Figure S2A,C). Phosphorylation of threonine 175 (Thr175) in SnRK1α1 and threonine 176 (Thr176) in SnRK1α2 can be achieved by both activating kinases. Furthermore, the phosphorylation of this threonine residue is essential for maintaining kinase activity (Shen et al. 2009; Maya‐Bernal et al. 2017; Figure S2A–D).

**FIGURE 1:**
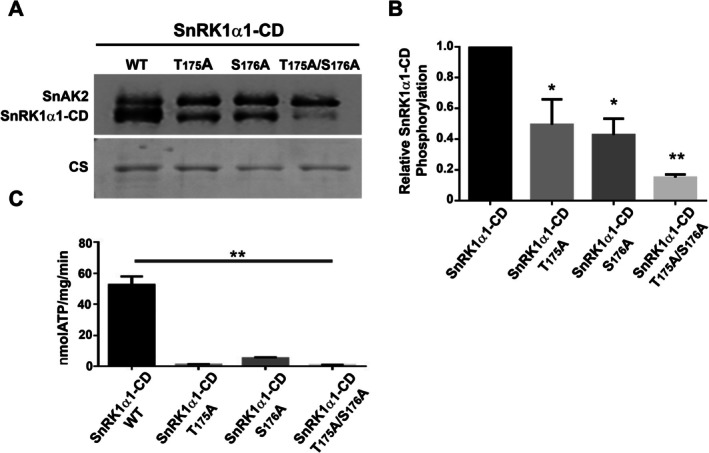
Phosphorylation of SnRK1α1 Catalytic Domain (CD) by SnAK2 kinase. (A) The incorporation of ^32^P into wild type (WT), T175A, S176A, or T175A/S176A mutant proteins was evaluated following SnAK2 kinase assays. The activation assay was conducted for 1 h at 30°C, as detailed in Material and methods section. Proteins were separated via SDS‐PAGE and transferred onto PVDF membranes. These membranes were then exposed to an Imaging Screen HD (Bio‐Rad), and the resulting images were captured using a Molecular Imager FX system (Bio‐Rad). (B) Densitometric analysis evaluating the comparative phosphorylation levels within the catalytic domain of WT SnRK1α1 and its mutants. (C) Kinase activity of the SnRK1α1 catalytic domain was quantified by measuring the incorporation of ^32^P into the AMARA peptide substrate after pre‐activation with SnAK2. The activity is expressed as nmol of ATP incorporated per mg of protein per minute (nmol ATP/mg/min). The results are the mean ± SD from three different experiments, with a *p*‐value of 0.05.

Experiments utilizing phosphomimetic mutants—where Thr175 in SnRK1α1 and Thr176 in SnRK1α2 were substituted with aspartic or glutamic acid—produced varying results depending on the experimental system used (Crozet et al. 2010; Avila et al. 2012; Cho et al. 2016; Maya‐Bernal et al. 2017; Figure 1). In our studies, these mutants continued to incorporate ^32^P at a site not recognized by the pThr175‐specific antibody, which can recognize the phosphorylation site of both SnRK1α1 and SnRK1α2. However, their activity against the AMARA peptide was significantly reduced compared with the WT (Figures 1 and S2). Since SnRK1α1 is responsible for most of the cellular activity (Zhang et al. 2009) and both activating kinases have identical effects, all subsequent experiments were conducted focusing on SnRK1α1 and SnAK2.

Recent phosphoproteomic analyses of Arabidopsis plants cultivated under different conditions have revealed Ser176 in SnRK1α1 as an additional phosphorylated residue (Umezawa et al. 2013; Zhang et al. 2013; Choudhary et al. 2015). To investigate if Ser176 can be phosphorylated by SnAK2 in T175A mutants, we performed ^32^P incorporation assays using various CD point mutants. Consistent with previous studies, the T175A mutation significantly reduced both ^32^P incorporation and kinase activity, emphasizing its critical role in SnRK1α1 activation (Baena‐González et al. 2007; Shen et al. 2009; Maya‐Bernal et al. 2017; Figure 1). Replacing Ser176 with Ala also caused a similar decrease in phosphorylation levels (Figure 1A,B) and significantly reduced kinase activity (Figure 1C). For the double mutants (T175A/S176A), kinase activity was lost even though ^32^P incorporation was still detectable. The observed residual signal likely represents autophosphorylation activity as previously reported for SnRK1α1 (Baena‐González et al. 2007). To verify this, a kinase‐dead SnRK1α1 CD with a K48A substitution was analyzed. This mutated enzyme exhibited significantly reduced phosphorylation when SnAK2 was present. Remarkably, the triple mutant K48A/T175A/S176A showed an almost complete absence of this signal, strongly indicating that SnAK2 mediates the phosphorylation of Thr175 and Ser176. Any residual phosphorylation within the CD appears to be the result of autophosphorylation (Figure S2E). In all instances, kinase activity remained minimal (Figure S2F).

### 3.2 Prediction of SnRK1α1's Three‐Dimensional Structure in Complex With AMARA Substrate

**FIGURE 2:**
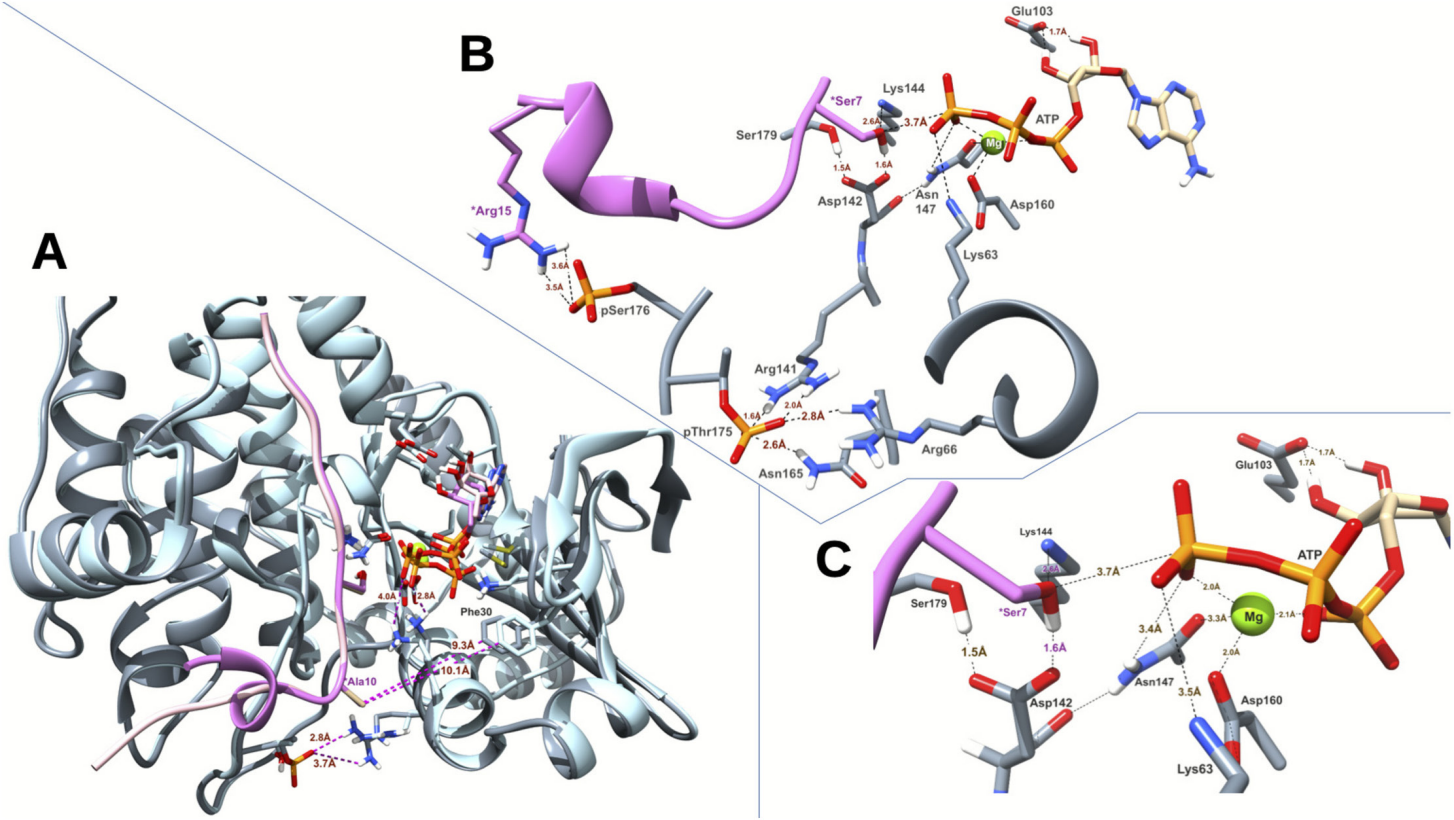
Three‐Dimensional structure of SnRK1α1 catalytic domain, in complex with MgATP and the AMARA peptide. (A) Superposition of the complex predicted with the unmodified enzyme (light blue) or the pThr175‐pSer176 (slate gray) form. The AMARA peptide is shown in light and darker pink. (B) Detail of the putative active‐site geometry of SnRK1α1‐pThr175‐pSer176 in complex with its substrates. The AMARA peptide is shown in orchid color and SnRK1α1 in slate gray. Only selected groups are shown. (C) Close‐up of the active site in (B) showing the predicted contacts possibly relevant to catalysis. Dashed lines indicate distances in Å. *Labels for AMARA residues.

The molecular basis underlying the double phosphorylation requirement of SnRK1α1 remains unclear. Figure 2 illustrates the overall predicted structure of the SnRK1α1 catalytic domain in complex with its substrates. This image represents a superposition of the predicted complexes for both unmodified SnRK1α1 and its doubly phosphorylated form, SnRK1α1‐pThr175‐pSer176. The presence of pThr175 is predicted to form a salt bridge with Arg66 and with Arg141 (Figure 2B), resulting in a slightly more compact protein conformation. For instance, the distance between Phe30 and Ala10 decreases from 10.1 Å to 9.3 Å, positioning Lys63 closer to the γ‐phosphate of ATP, reducing the distance from 4 Å to 2.8 Å (Figure 2A,C). In addition, the predicted structure further offers a plausible explanation for the necessity of double phosphorylation: pSer176 establishes contact with the AMARA peptide via the side chain of Arg15 (Figure 2B).

Phosphomimetic mutations, where Thr175 or Ser176 are substituted with Asp, do not result in permanent activation of the enzyme (this work; Maya‐Bernal et al. 2017). Consistent with model predictions, replacing Thr175 with Asp likely produces a protein with insufficient negative charge density to effectively counterbalance two positive charges simultaneously. This deficiency may explain the observed reduction in enzyme activity (Figure 2B).

The changes in the enthalpy of the complexes when the AMARA peptide is removed are shown in Table S2. The ∆H values reveal stronger contacts of AMARA with the unmodified SnRK1α1 than with the phosphorylated forms. In fact, SnRK1α1‐pThr175‐pSer176 has the highest energy of interaction with the AMARA and the second lowest with AMARA^pSer7^.

### 3.3 Functional Role of pSer176 in SnRK1α1

**FIGURE 3:**
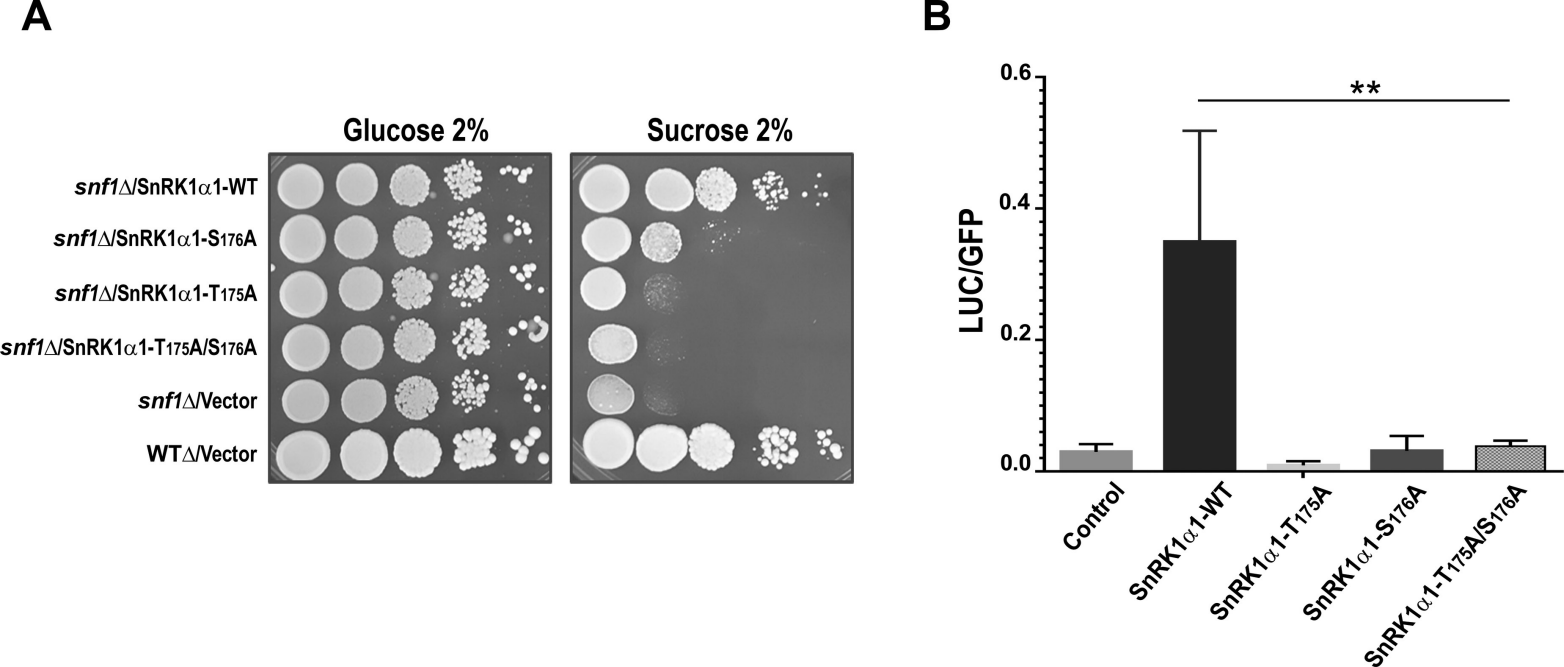
Impact of pSer176 on SnRK1α1 Activity. (A) Wild‐type (WT) SnRK1α1 and its mutants—T175A, S176A, and T175A/S176A—were introduced into the Δ*snf1* strain. Transformants were plated at an OD600 of 1.0 (10^0^), followed by the preparation of four 10‐fold serial dilutions (10^−1^ to 10^−4^). These dilutions were then spotted from left to right. Growth patterns were observed after incubating the cultures for 3 days at 30°C. (B) WT SnRK1α1 and the T175A, S176A, and T175A/S176A mutants were transiently expressed in Arabidopsis protoplasts, together with the *pDIN*6::*Luciferase* reporter gene as an indicator of kinase activity and the *35S::GFP* as the transfection control. Control protoplasts were only transformed with *35S::GFP* and *pDIN*6::*Luciferase*. The data represent the mean ± standard deviation (SD) from two independent biological experiments, each performed with three technical replicates, with a *p*‐value of 0.05.

To evaluate the functional role of Ser176 phosphorylation in the SnRK1α1 subunit, we used the *snf1Δ* yeast mutant, which is unable to grow on media containing alternative carbon sources such as sucrose. This strain was complemented with different full‐length *SnRK1α1* variants. As expected, transformation with wild‐type *SnRK1α1* restored growth on sucrose‐containing media. However, yeast transformed with the T175A mutant or the double mutant T175A/S176A did not grow, confirming that phosphorylation at the canonical Thr175 is essential for activity. Interestingly, the S176A single mutant also failed to fully restore the wild‐type growth phenotype (Figure 3A), confirming the requirement of the Thr175/Ser176 pair for enzyme function. In addition to the recombinant protein activity assays and yeast complementation experiments, the functional role of Ser176 phosphorylation was evaluated in Arabidopsis protoplasts using the *pDIN6::LUC* reporter gene as a readout of SnRK1 activity (Baena‐González et al. 2007). The results showed that only the wild‐type version of SnRK1α1 activated luciferase expression, whereas the T175A, S176A, and the double mutant T175A/S176A versions failed to induce any luciferase activity (Figure 3B). These findings further support the importance of Ser176 phosphorylation in SnRK1α1 activity.

### 3.4 Analysis of SnRK1α1 Activity and Phosphorylation Patterns Throughout the Day and During Prolonged Darkness

To evaluate the activity and phosphorylation status of SnRK1α1, we developed highly specific antibodies targeting distinct phosphorylation sites. The specificity of these antibodies in differentiating the phosphorylation states at each site was confirmed using ELISA assays (Figure S3A).

In western blot analyses using plant extracts, the antibodies designed to recognize pThr175 successfully detected two distinct protein bands, corresponding to SnRK1α1 and SnRK1α2. These results were consistent with previously reported data (Baena‐González et al. 2007) and were further validated using *snrk1α1* and *snrk1α2* mutant samples (Figure S3B). The pSer176 antibody detected not only a band at the expected molecular weight but also an additional lower molecular weight band, which could represent a degradation product. This possibility is supported by the observation that both pSer176‐associated bands appear in *snrk1α1* and *snrk1α2* mutant backgrounds, suggesting that either isoform is recognized as two distinct bands (Figure S3B).

**FIGURE 4:**
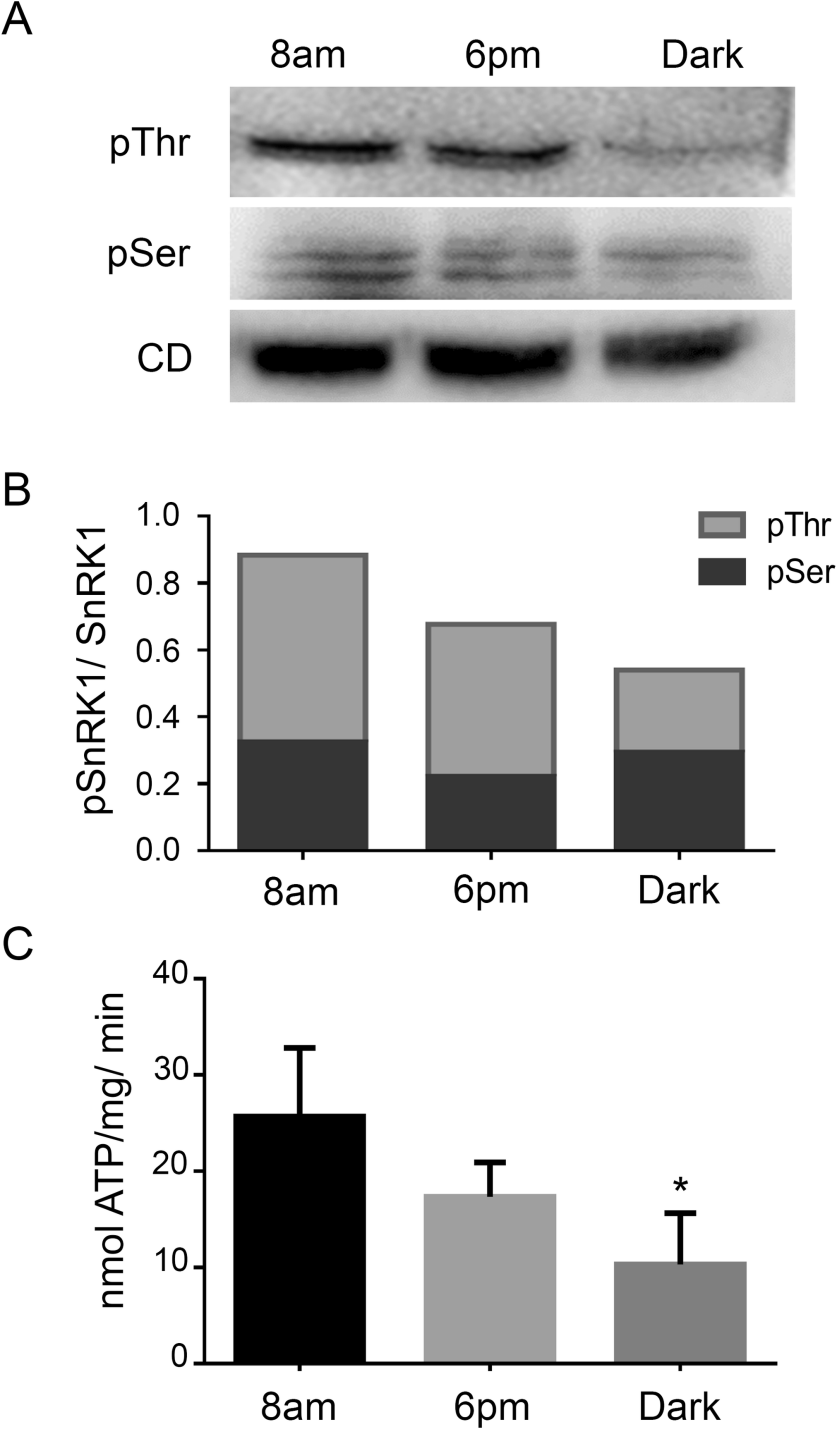
Phosphorylation of Thr175 and Ser176 in SnRK1α1 at various time points during the day/night cycle. (A) Antibodies specific to phosphorylated Thr175 (pThr175), phosphorylated Ser176 (pSer176), and the catalytic domain (CD) were utilized to assess the levels of phosphorylated and non‐phosphorylated SnRK1α1. Plant samples were harvested at three key time points: 8 am, 6 pm, and after 24 h of extended darkness. (B) Quantitative analysis was performed on the protein bands shown in panel A to compare the relative abundance of phosphorylated versus non‐phosphorylated SnRK1α1. (C) Kinase activity assays were conducted using plant extracts collected at the same time points mentioned in panel B to evaluate the functional impact of phosphorylation. The data represent the mean ± standard deviation (SD) derived from two independent biological experiments, each with four technical replicates. Asterisks (*) denote statistically significant differences with a *p*‐value of 0.05.

A comparative analysis of the phosphorylation levels at Thr175 and Ser176 relative to the catalytic domain revealed distinct temporal patterns in WT plants. Thr175 phosphorylation peaked at 8 am, showed a slight decrease by 6 pm, and dropped significantly during prolonged darkness. In contrast, Ser176 phosphorylation increased after 8 am and remained stable throughout the subsequent period (Figure 4A).

To elucidate the importance of Thr175 and Ser176 phosphorylation in relation to SnRK1 activity, the ratios of pThr175 and pSer176 to the catalytic subunit were calculated (Figure 4B). The data revealed that pThr175 phosphorylation exhibited a stronger correlation with kinase activity fluctuations, peaking at the onset of the light phase (8 am) and significantly declining by 6 pm and after 24 h of continuous darkness (Figure 4C).

### 3.5 SnRK1α1 Double Variant T175A/S176A Accumulates at the Cytoplasm During Heat Stress

**FIGURE 5:**
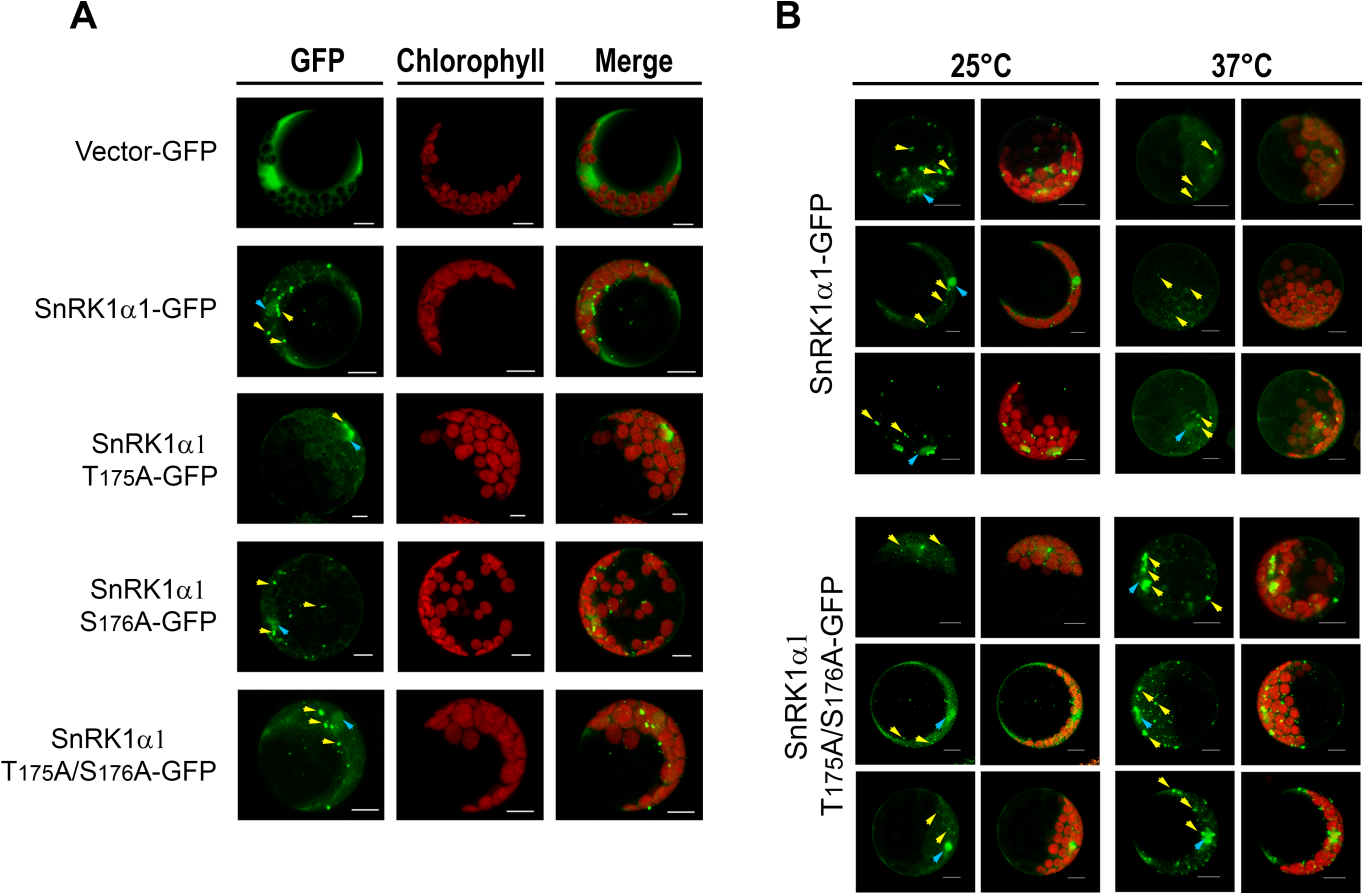
Subcellular Localization of WT, T175A, S176A, and T175A/S176A SnRK1α1 Proteins. (A) The wild‐type (WT) SnRK1α1 protein, along with various unphosphorylatable mutant forms, was transiently expressed in Arabidopsis protoplasts as GFP fusion proteins. Following the incubation period, these proteins were visualized using a Leica TCS‐SP8 confocal microscope to determine their subcellular localization. (B) Arabidopsis protoplasts transfected with WT and T175A/S176A mutant versions of SnRK1α1 were incubated at either 25°C or 37°C for 1 h. Confocal microscopy images were taken to evaluate how temperature affects protein localization. The scale bar represents 10 μm. Yellow arrows highlight cytoplasmic aggregates, and blue arrows indicate nuclear aggregates.

To assess the impact of phosphorylation loss on subcellular localization, we transiently expressed wild‐type (WT) SnRK1α1 fused to GFP in protoplasts alongside its T175A, S176A, and T175A/S176A mutants. Confocal microscopy analysis demonstrated that both WT and mutant variants localized within the cytoplasm and nucleus, with noticeable formation of small cytoplasmic aggregates (Figure 5A).

Significantly, when protoplasts were subjected to heat stress at 37°C, the number of cytoplasmic aggregates in WT SnRK1α1 decreased, indicating their degradation. In contrast, these aggregates persisted and even accumulated in the cytoplasm of the double mutant. This finding suggests a relevant role for phosphorylation in promoting the degradation or movement of cytoplasmic aggregates, particularly under stress conditions (Figure 5B).

## 4 Discussion

### 4.1 Phosphorylation of Ser176 on SnRK1α1

Given the significant role played by SnRK1α1 activity in plant growth and development, understanding some of the mechanisms behind its activation is essential. It is widely believed that phosphorylation at specific threonine residues—Thr175 in SnRK1α1 and Thr176 in SnRK1α2—is the primary post‐translational modification responsible for SnRK1 activation (Shen et al. 2009). However, as with many kinases, other phosphorylation sites may also significantly influence this activation (Aslam and Ladilov 2022).

Recent research using phosphoproteomic techniques on Arabidopsis plants grown in different conditions has revealed new phosphorylated residues. These include Ser176, Ser179, and Ser366 in SnRK1α1, and Ser177 in SnRK1α2 (Umezawa et al. 2013; Zhang et al. 2013; Choudhary et al. 2015; Al‐Momani et al. 2018). Although some of these findings require further confirmation, they provide critical insights for exploring additional phosphorylation sites and their role in kinase activation. In our in vitro experiments with the catalytic domain of SnRK1α1, we observed the incorporation of ^32^P into Thr175 and Ser176, dependent on SnAK2 kinase activity. Mutant versions of the catalytic domain, where Thr175 and Ser176 were replaced with alanine, displayed significantly lower activity compared with the wild type. The structural modeling of the SnRK1α1 catalytic domain, in complex with its reaction products (MgADP and the AMARA peptide phosphorylated at Ser7), enables us to hypothesize about the need for such dual phosphorylation. The model suggests that phosphorylated Ser176 interacts with the AMARA peptide through Arg15, while phosphorylated Thr175 forms connections with both Arg141 and Arg66. This implies that natural targets of SnRK1α1, such as FUS3 (Tsai and Gazzarrini 2012), IDD8 (Jeong et al. 2015), and HMGR1S (Robertlee et al. 2017), possess two or more basic residues near their serine phosphorylation sites. These basic residues likely enhance their binding affinity with the enzyme's phosphorylated residues, facilitating efficient interactions. Mutating these sites and assessing their interaction with the kinase would be highly interesting.

Furthermore, this structural arrangement may explain why mutating Thr175 to aspartic acid disrupts kinase activity. The mutation fails to maintain the necessary negative charge density to balance the positive charges of the arginine residues.

Thermodynamic analyses further support these findings. Changes in enthalpy (∆H), presented in Table S2, showed a stronger binding of the AMARA peptide to the unphosphorylated SnRK1α1 than to its phosphorylated forms. Interestingly, the SnRK1α1 variant with both pThr175 and pSer176 exhibits the highest interaction energy with AMARA and a significantly reduced interaction with AMARA^pSer7^. While this might seem counterintuitive, tighter substrate binding requires more energy to reach the transition state, leading to a slower reaction rate. Unfortunately, we could not assess entropy changes for these complexes due to the limitations of the theoretical approach used (SQM‐PM7‐LMO), which currently cannot handle thermochemical calculations for large molecular systems.

A potential chemical mechanism of catalysis may be proposed from the model; yet, it is not discussed here because it is beyond the scope of the current work.

### 4.2 Functional Effect on pSer176

The in vivo activity of SnRK1α1 was evaluated using a complementation assay in a ∆*snf1* mutant yeast strain. In this experimental setup, only the wild‐type (WT) SnRK1α1 was able to support yeast growth on sucrose‐containing media, highlighting the critical importance of phosphorylation at Thr175 for functional activity. Notably, the S176A mutant was unable to complement the mutant strain, reinforcing the critical importance of phosphorylation at this site. These yeast complementation results were corroborated by transient expression experiments conducted in Arabidopsis protoplasts. In these assays, both WT and mutant versions of SnRK1α1 were assessed for kinase activity using the *pDIN6*::*luciferase* reporter gene as an indicator of kinase function (Baena‐González et al. 2007). The findings from both yeast and plant systems consistently demonstrated that only the phosphorylated WT protein retained functional activity. When comparing these phosphorylation dynamics to homologous sites in related kinases, distinct regulatory behaviors were observed. In AMPK, phosphorylation of the Ser173 residue negatively regulates the kinase by inhibiting the phosphorylation and activation of Thr172 (Aslam and Ladilov 2022). Conversely, in the SNF1 kinase, mutation of Ser211 to alanine had no discernible effect on kinase activity. Apparently, divergent regulatory mechanisms have emerged within the AMPK/SNF1/SnRK1 kinase family (Martínez‐Barajas and Coello 2019).

### 4.3 Correlation of Kinase Activity and Stability With Thr175 and Ser176 Phosphorylation in SnRK1α1

Our study demonstrated a peak of SnRK1 enzyme activity at 8 am, with a tendency to decline by the evening. This pattern closely aligns with phosphorylation at the Thr175 site, which was also most prominent at 8 am, decreasing steadily thereafter (Figure 4). Conversely, phosphorylation at the pSer176 site remained relatively stable during the same timeframe.

Interestingly, the unphosphorylated catalytic subunit consistently shows high levels, indicating that only a small fraction undergoes phosphorylation. While SnAKs are the only kinases known to activate SnRK1 via Thr175 phosphorylation (Glab et al. 2017), these have been identified in extracts from young plants, but not in expanding, mature, or senescent plants—despite the presence of pThr175 in the catalytic subunit. This suggests the possible involvement of another kinase (Shen et al. 2009). Furthermore, the lack of documented information on Ser176 phosphorylation highlights a significant area for further research. Key questions to explore include whether Ser176 is the first site to undergo modification, thereby maintaining a stable pool throughout the day, or if it is phosphorylated simultaneously with Thr175. Investigating these possibilities could provide valuable insights into this critical issue.

In living organisms, SnRK1 activity is modulated by several factors, including sugar phosphates, trehalose‐6‐phosphate (T6P), the circadian clock, and various downstream regulatory components (Avidan et al. 2023). One such downstream regulator could be protein degradation, a known mechanism for controlling SnRK1 levels. According to Sun et al. (2024), following phosphorylation and activation at Thr175, SnRK1 undergoes degradation through a SnAKs‐mediated pathway. This complex process of protein turnover may explain the observed differences in phosphorylation levels between pThr175 and pSer176, suggesting that phosphorylation at one or both sites might tag the kinase for degradation.

Our experimental results reveal a potential link between SnRK1α1 phosphorylation and its degradation. In transient expression assays with non‐phosphorylatable mutants fused to GFP, we consistently observed cytoplasmic granules, even under stress conditions. Notably, the phosphorylation state of SnRK1α1 did not influence its subcellular localization, as all mutant variants were present in both the nucleus and cytoplasm. Under heat stress, non‐phosphorylatable SnRK1α1 double mutant exhibited prominent cytoplasmic aggregates. This suggests a strong association between phosphorylation status and protein stability, highlighting the critical role of phosphorylation in regulating not only kinase activity but also the stability of active proteins. Furthermore, it has been shown that SnRK1α1 interacts with Tudor *Staphylococcus* nuclease (TSN) following heat shock stress, promoting stress granule (SG) formation and enhancing its activity (Gutierrez‐Beltran et al. 2021). Further research is necessary to explore the role of SGs in SnRK1α1 activity and to clarify the precise mechanisms that prevent prolonged kinase activation.

## Author Contributions

Alejandra Ávila, Eleazar Martínez, and Patricia Coello designed the experiments. Alejandra Ávila, Aitana López, and Patricia Coello performed the experiments. Rogelio Rodríguez modeled the SnRK1 α1 structure. Alejandra Ávila, Eleazar Martínez, Rogelio Rodríguez, and Patricia Coello wrote the manuscript. Alejandra Ávila, Eleazar Martínez, and Patricia Coello discussed the manuscript. All authors reviewed and approved the final version of the manuscript.

## Funding

This work was supported by Dirección General de Asuntos del Personal Académico, Universidad Nacional Autónoma de México, IN201922.

## Conflicts of Interest

The authors declare no conflicts of interest.

## Supporting information


**Data S1:** Supplementary Information.

## Data Availability

The data that support the findings of this study are available from the corresponding author upon reasonable request.
