# Supplementary material for: Dual Phosphorylation of Thr175 and Ser176 Is Essential for SnRK1α1 Activation
Source: Physiol Plant. 2025 Dec 29;178(1):e70726. doi: 10.1111/ppl.70726 (PMC12746220; doi:10.1111/ppl.70726)
Supplement: Supplementary file 1 — Data S1: Supplementary Information. [file PPL-178-e70726-s001.pdf]

Dual phosphorylation of Thr175 and Ser176 is essential for SnRK1α1 activation

Alejandra Ávila[1], Aitana López[1], Jacquelynne Cervantes[2], Rogelio Rodríguez-Sotres[1], Eleazar Martínez-Barajas[1] and Patricia Coello[1*]

Supplementary Figure 1. CRISPR-Cas9 editing of *SnRK1α1* and *SnRK1α2*. The CRISPR-Cas9 system was utilized to target exon 2 of both *SnRK1α1* and *SnRK1α2* catalytic subunits, as outlined in the Materials and Methods section. The editing process involved the insertion of an additional nucleotide, which disrupted the open reading frame and resulted in the introduction of a premature stop codon. The black asterisk (*) marks the location of the extra T insertion, whereas the red asterisk (*) denotes the premature introduction of a stop codon in SnRK1α1 and SnRK1α2.

Supplementary Figure 2. Phosphorylation and activation of SnRK1α1 and SnRK1α2 Catalytic Domains (CD) by SnAKs. (**A and C)** The incorporation of radioactive phosphate (^{32}P) into the CD of SnRK1α1 and SnRK1α2 was analyzed after incubation with SnAK1 and SnAK2. Both wild-type (WT) and T175D/T176D mutants of SnRK1α1 and SnRK1α2, respectively, were examined. Phosphorylation at threonine residues 175/176 (Thr175/Thr176) was specifically detected using anti-pT175/176 antibodies, confirming phosphorylation in WT samples but not in the Thr175/176D mutants. WT samples for both kinases were analyzed in duplicate, with SnAKs alone (also in duplicate) serving as controls. Coomassie staining (CS) was used as a loading control. **(B and D)** The kinase activities of the WT and T175/176D mutants of SnRK1α1 and SnRK1α2 were evaluated following activation by SnAKs kinases, highlighting activity variations influenced by

phosphorylation status. SnAK1 and SnAK2 individually functioned as control groups. **(E and F)** Catalytically inactive SnRK1α1 CD with a K48A mutation and a triple mutant (K48A/T175A/S176A) were incubated with or without SnAK2. ^{32}P incorporation and activity against the AMARA peptide were assessed. Data represent the mean ± SD from three independent experiments, with a significance threshold of p-value of 0.05.

Supplementary Figure 3. Specific antibodies targeting pThr175 and pSer176. **(A)** ELISA Assays demonstrating antibody specificity. Closed triangles indicate the strong specificity between the pSer antibody and the pSer peptide (▲), while closed dots represent the specificity of pThr antibodies towards the pThr peptide (●). Inverted triangle and dotted line pattern illustrate the cross-reactivity between the pSer antibody and the pThr peptide (▼), and square with dotted line pattern show the cross-reactivity between the pSer antibody and the pThr peptide (■). **(B)** Arabidopsis SnRK1 was identified using antibodies against pThr175, pSer176 and anti-CD in WT, snrk1*α1*, and snrk1*α2* Arabidopsis plants. The samples were collected at the start of the day (8 AM) to ensure consistency in protein expression analysis.

S1

SnRK1α1
(At3g01090.1)

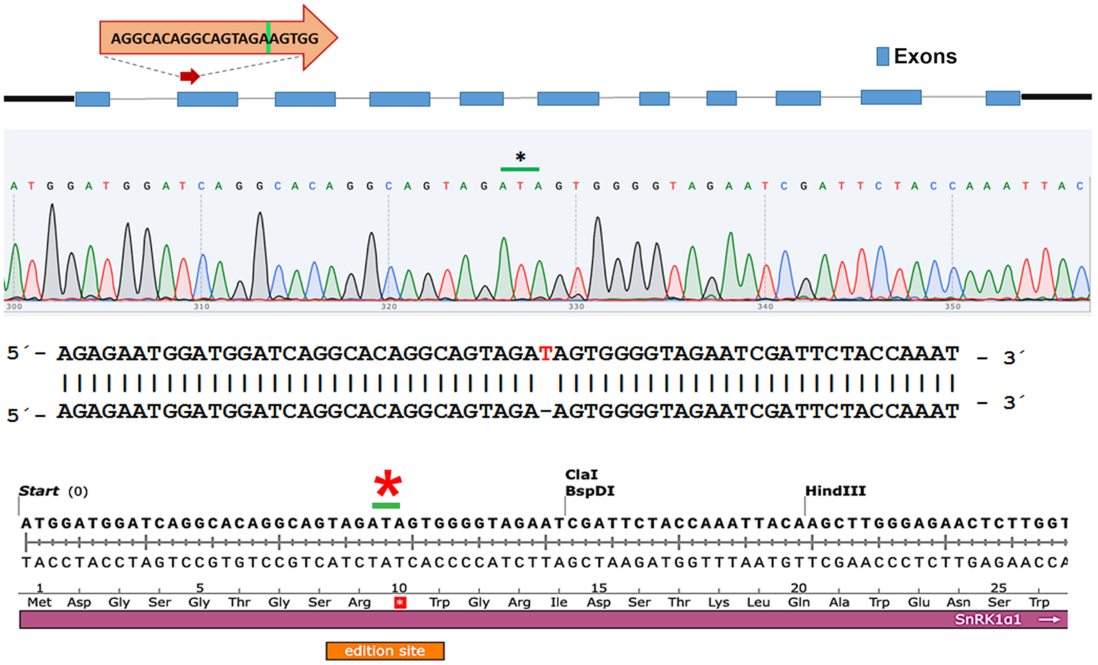

SnRK1α2
(At3g29160.1)

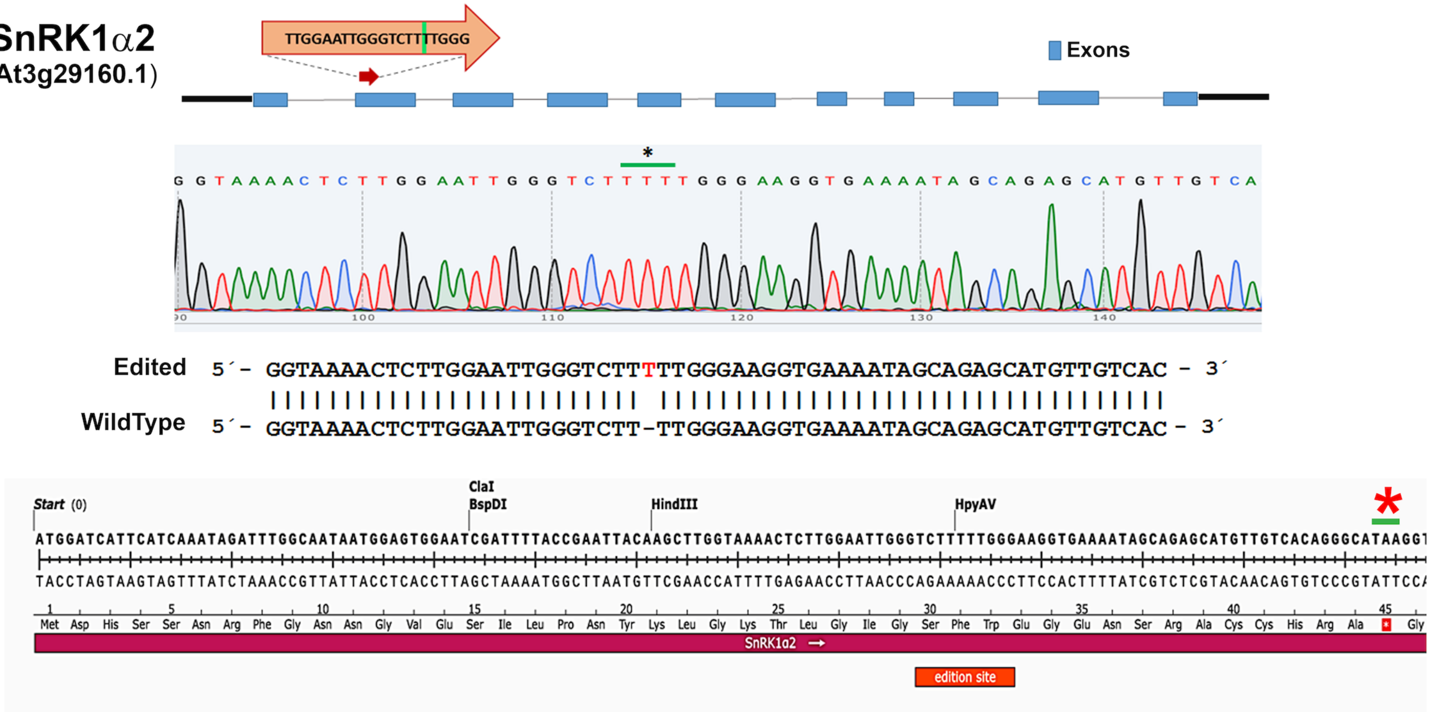

S2

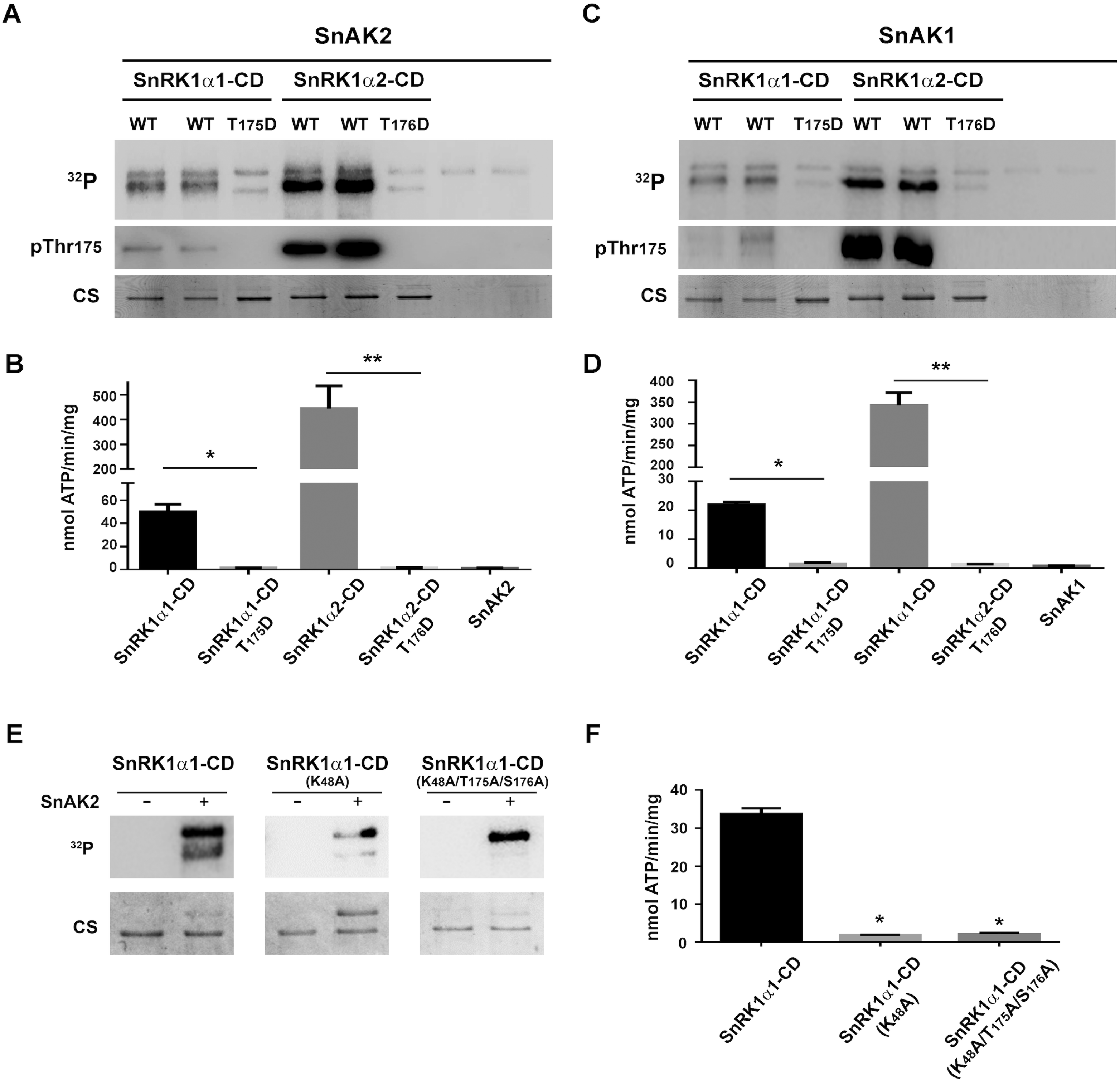

A
SnAK2
SnRK1α1-CD
SnRK1α2-CD
WT
WT
T175D
WT
WT
T176D
32P
pThr175
CS
C
SnAK1
SnRK1α1-CD
SnRK1α2-CD
WT
WT
T175D
WT
WT
T176D
32P
pThr175
CS
B
nmol ATP/min/mg
500
400
300
200
60
40
20
0
*
**
SnRK1α1-CD
SnRK1α1-CD T175D
SnRK1α2-CD
SnRK1α2-CD T176D
SnAK2
D
nmol ATP/min/mg
400
350
300
250
200
30
20
10
0
*
**
SnRK1α1-CD
SnRK1α1-CD T175D
SnRK1α1-CD
SnRK1α2-CD T176D
SnAK1
E
SnRK1α1-CD
SnRK1α1-CD (K48A)
SnRK1α1-CD (K48A/T175A/S176A)
SnAK2
-
+
-
+
-
+
32P
CS
F
nmol ATP/min/mg
40
30
20
10
0
*
*
SnRK1α1-CD
SnRK1α1-CD (K48A)
SnRK1α1-CD (K48A/T175A/S176A)

S3

A

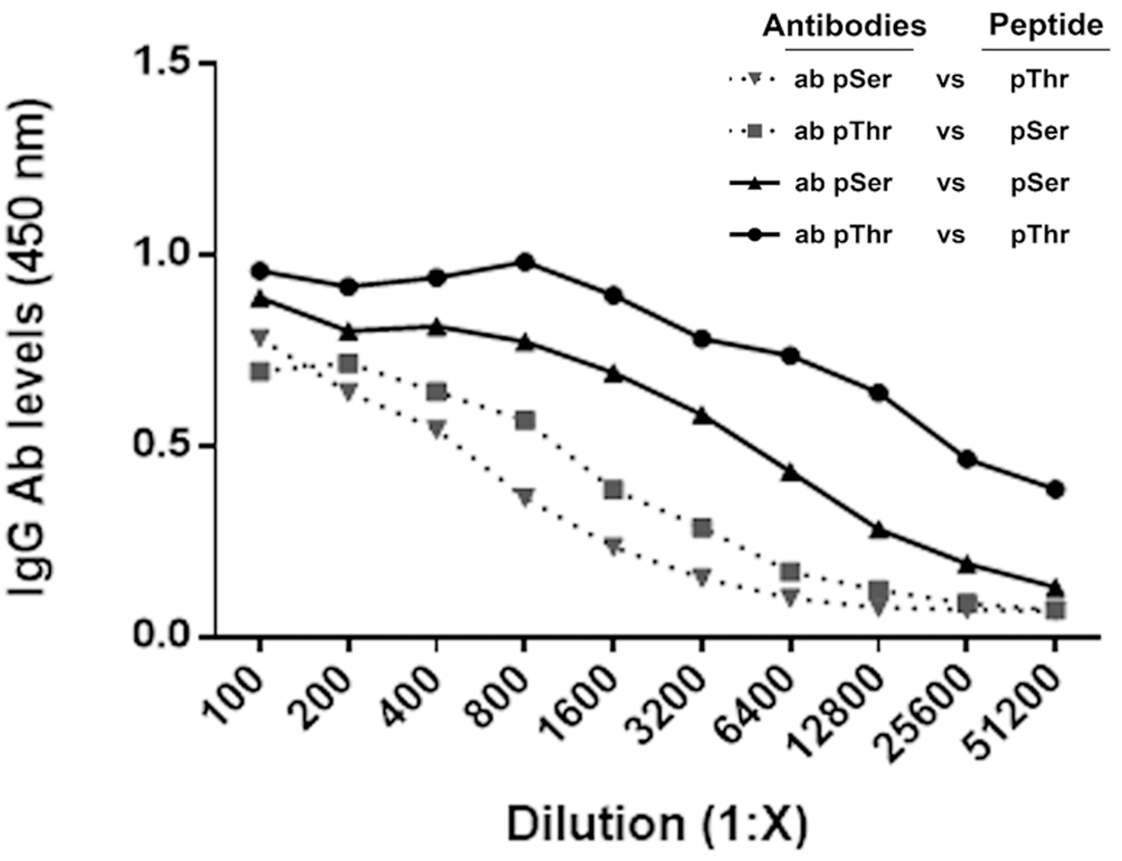
Antibodies
Peptide
ab pSer vs pThr
ab pThr vs pSer
ab pSer vs pSer
ab pThr vs pThr
IgG Ab levels (450 nm)
1.5
1.0
0.5
0.0
100
200
400
800
1600
3200
6400
12800
25600
51200
Dilution (1:X)

B

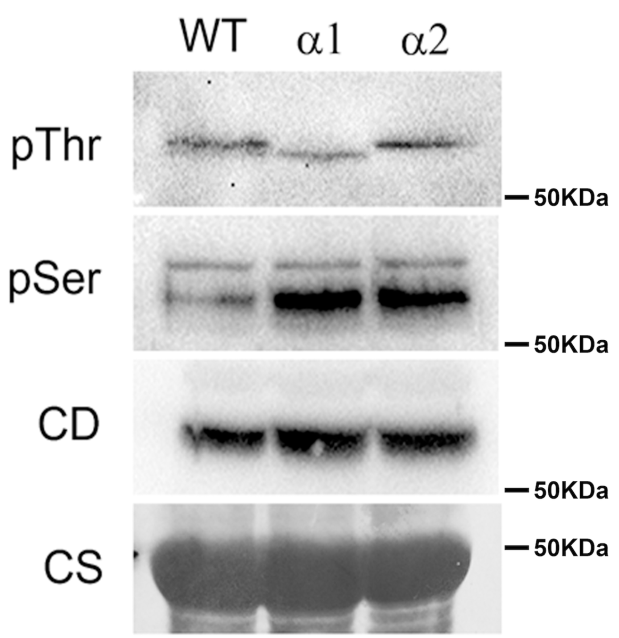
WT
α1
α2
pThr
pSer
CD
CS
50KDa
50KDa
50KDa
50KDa

Supplementary Table 1. Primers sequences

Primer	Sequence 5´-3´
**SnRK1α1 (1-341) FWD**	CGGGATCCATGGATGGATCAGGC
**SnRK1α1 (1-341) REV**	CGGGAATTCTCAATAACCACTAGAGGC
**SnRK1α2 (1-342) FWD**	CGGGATCCATGGATCATTCATCA
**SnRK1α2 (1-342) REV**	CGGGAATTCTCAATAGCCACTTGGAAC
**SnRK1α1 (T175-A) midsense**	GATGGTCATTTTTTGAAGGCGAGTTGTGGAAGTCCA
**SnRK1α1 (T175-A) midantisense**	TGGACTTCCACAACTCGCCTTCAAAAAATGACCATC
**SnRK1α1 (S176-A) midsense**	GATGGTCATTTTTTGAAGACAGCGTGTGGAAGTCCA
**SnRK1α1 (S176-A) midantisense**	TGGACTTCCACACGCTGTCTTCAAAAAATGACCATC
**SnRK1α1 (TS-AA) midsense**	GATGGTCATTTTTTGAAGGCGGCGTGTGGAAGTCCA
**SnRK1α1 (TS-AA) midantisense**	TGGACTTCCACACGCCGCCTTCAAAAAATGACCATC
**DsREd A10bamut BsrG1 fwd**	GGGTGTACAAGATGGATGGATCAGGC
**NotIRevA10**	GCGCGGCCGCTCAGAGGACTCGGAGCTGAGC
**SnAK1 FWD**	GGGGAATTCCCATGTTTCGTGATAGTTTT
**SnAK1 REV**	GGGCTCGAGTTAGTTAGGATCTGAGGT
**SnAK2 FWD**	GGGGAATTCCCATGTTTTGTGATAGTTTT
**SnAK2 REV**	CCCCTCGAGTCAGCTATGGTTTTGATC
**mEGFPRevNotstop**	CTGAGCGGCCGCCTAACCTGACTTGTACAGCTCGTCC
**eGFP_Fw_BamHI**	CACAGGATCCATGGTGAGCAAGGG
**A10RevKpnNot**	GGGGCGGCCGCGACTCGGAGGGTACCGAGGACTCGGAGCTGAGCAAGAAA
**SnRK1α1 (guide RNA)**	AGGCACAGGCAGTAGAAGTGG
**SnRK1α2 (guide RNA**	TTGGAATTGGGTCTTTTGGGA

Supplementary Table 2. Theoretical Binding Enthalpies AMARA and AMARApSer7 peptides in SnRK1α1-MgADP-AMARApSer7 (Enzyme-products) and SnRK1α1-MgATP-AMARA (Enzyme-substrate) complexes

Complex	MgADP ΔH (kCal/mol)†	+	AMARA pSer7 Δsurf (nm^2)‡	MgATP ΔH (kCal/mol)	+	AMARA Δsurf (nm^2)
**SnRK1α1**	-125.4 (-11.9)		-12.5	-115.3 (-11.1)		-12.1
**SnRK1α1-pThr175**	-150.2 (-14.3)		-10.4	-106.2 (-10.3)		-9.96
**SnRK1α1-pSer176**	-78.2 (-7.42)		-12.1	-92.37 (-8.92)		-11.6
**SnRK1α1-pThr175-pSer176**	-92.8 (-8.80)		-12.3	-85.9 (-8.30)		-11.9
